# PTP10D-mediated cell competition is not obligately required for elimination of polarity-deficient clones

**DOI:** 10.1242/bio.059525

**Published:** 2022-11-10

**Authors:** Stephan U. Gerlach, Geert de Vreede, David Bilder

**Affiliations:** Department of Molecular and Cell Biology, University of California-Berkeley, Berkeley, CA 94720, USA

**Keywords:** *Drosophila*, Cell competition, Epithelial polarity, Scrib, Dlg, PTP10D

## Abstract

Animal organs maintain tissue integrity and ensure removal of aberrant cells through several types of surveillance mechanisms. One prominent example is the elimination of polarity-deficient mutant cells within developing *Drosophila* imaginal discs. This has been proposed to require heterotypic cell competition dependent on the receptor tyrosine phosphatase PTP10D within the mutant cells. We report here experiments to test this requirement in various contexts and find that PTP10D is not obligately required for the removal of *scribble* (*scrib*) mutant and similar polarity-deficient cells. Our experiments used identical stocks with which another group can detect the PTP10D requirement, and our results do not vary under several husbandry conditions including high and low protein food diets. Although we are unable to identify the source of the discrepant results, we suggest that the role of PTP10D in polarity-deficient cell elimination may not be absolute.

## Teaser

Summary: In contrast to previous mechanistic models about heterotypic cell competition, not all experimental conditions require PTP10D to eliminate polarity-deficient clones.

## INTRODUCTION

Developing tissues need to assess whether cells within them have been properly produced, and so have developed several types of mechanisms to eliminate inappropriate cells. Initially studied in *Drosophila* epithelia, mechanisms including cell competition, cell extrusion, and extrinsic cell elimination have also been demonstrated in mammalian tissues *in vivo* as well as in cell culture (Amoyel and Bach, 2014; Johnston, 2014; Madan et al., 2018; Vishwakarma and Piddini, 2020). In addition to different growth rates and different cell fates, altered cell polarity has long been recognized as a parameter detected by *Drosophila* imaginal discs that results in apoptotic elimination. Clones of cells mutant for the core polarity-regulating genes *scribble* (*scrib*), *discs-large* (*dlg*) and *lethal giant larvae* (*lgl*) that are generated in larval imaginal discs are killed and do not contribute to the adult tissue, instead being replaced by wild-type (WT) cells (Brumby and Richardson, 2003; Morata and Calleja, 2020; Nagata and Igaki, 2018; Uhlirova et al., 2005). Extensive studies have shown that apoptosis is driven by JNK signaling within the polarity-deficient cell, driven by the *Drosophila* TNF ligand Eiger (Egr) (Andersen et al., 2015; De Vreede et al., 2022; Igaki et al., 2009; La Marca and Richardson, 2020; Nagata and Igaki, 2018). A challenge has been to understand how the loss of cell polarity is sensed to activate apoptotic signaling in these cases.

A model by which polarity-deficient clones can be eliminated by cell competition was put forth in 2017 by Yamamoto et al., who identified a role for heterotypic cell interactions driving *scrib* cell elimination (Yamamoto et al., 2017). Briefly, the model proposes that upon polarity loss, two normally apically localized proteins come together at the clone boundary. The receptor tyrosine phosphatase PTP10D in mispolarized mutant cells binds the transmembrane protein Stranded at second (Sas) in WT neighbors whose own polarity is altered where they contact mutant cells, presumably because of loss of cell–cell junctions. Sas serves as a ligand to activate PTP10D at the border of polarity-deficient clones, leading to inhibition of EGFR signaling in the cell. EGFR inhibition allows Egr/JNK signaling to activate apoptosis in the clone, but if EGFR is not inhibited – for instance, when PTP10D in *scrib* clones is absent – then Egr/JNK instead drives overproliferation to form neoplastic tumors (Yamamoto et al., 2017).

We have recently shown that the source of Egr that eliminates polarity-deficient clones in fly imaginal tissue is the fat body, rather than the disc cells or hemocytes (De Vreede et al., 2022). In WT animals, fat body-produced Egr present in circulation is physically segregated from its receptor Grindelwald (Grnd), which is localized in imaginal discs exclusively at the apical surface. However, when a cell loses polarity*,* it mislocalizes Grnd to the basal surface, where Grnd binds to Egr and triggers activation of JNK signaling and apoptosis. This mechanism is driven by the autonomous polarized status of the mutant cell and is agnostic to the genotype of its neighbors. For instance, Egr from the fat body also binds to mispolarized Grnd and induces apoptosis in polarity-deficient cells when no WT cells are present.

Below, we describe results of experiments to investigate the role of PTP10D in elimination of *scrib* and other polarity-deficient clones. We were unable to identify conditions in which PTP10D is required for the elimination of such cells. This stands in contrast to both Yamomoto et al. and a second group, who have recently replicated the result that PTP10D loss increases the survival of *scrib* clones (Liu et al., 2022). The latter group provided identical stocks and shared details of food recipes and culture conditions with our own group, but consistent results could not be achieved in our hands. Although we have failed to identify the source of the inconsistency, we believe these data may be valuable for others investigating the role of PTP10D and heterotypic cell interactions in elimination of *scrib* cells.

## RESULTS AND DISCUSSION

### PTP10D depletion does not always rescue elimination of polarity-deficient clones

**Fig. 1.**
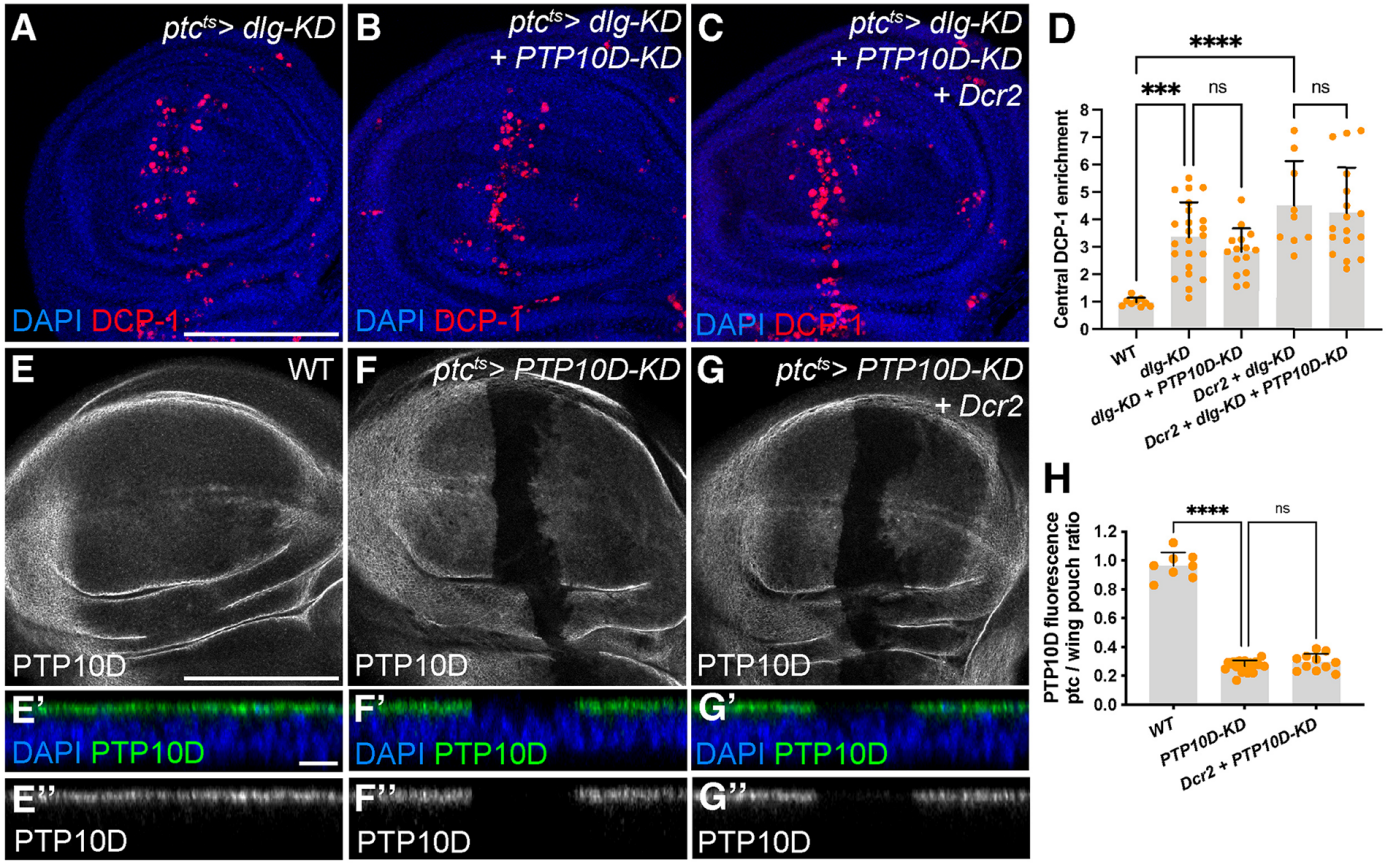
**PTP10D depletion does not alter removal of Dlg-deficient cells.** (A-D) *ptc-Gal4*-driven *dlg-KD* causes apoptosis along the A-P boundary (A; anti-DCP-1 in red) and additional *PTP10D-KD* does not alter apoptosis (B). Expression of Dcr2 along with *dlg-KD* triggers additional apoptosis due to enhancement of the long-inverted repeat RNA targeting *dlg*, but additional *PTP10D-KD* does not alter apoptosis (C). Quantitation in D (mean±s.d., one-way ANOVA test, *n*=9 for WT, *n*=23 for *dlg-KD*, *n*=15 for *dlg-KD+PTP10D-KD*, *n*=9 for *Dcr2+dlg-KD*, *n*=17 for *Dcr2+dlg-KD+PTP10D-KD*). (E-H) *PTP10D-KD* along the A-P boundary leads to strong reduction of PTP10D protein (F; control in E; anti-PTP10D in gray). Additional expression of Dcr2 does not lead to a stronger depletion of PTP10D (G). Quantitation in H (mean±s.d., one-way ANOVA test, *n*=8 for WT, *n*=19 for *PTP10D-KD*, *n*=11 for *Dc2+PTP10D-KD*). Scale bars: 100 µm in A, and E, 10 µm in E’. Statistical significance is indicated with *P≤0.05, **P≤0.01, ***P≤0.001, and ****P≤0.0001.

We revisited the role of PTP10D, whose depletion was reported to reduce elimination of *scrib* and *dlg* clones surrounded by WT cells when induced by *eyFLP*-driven mitotic recombination. We started by depleting the polarity regulator Dlg along the AP boundary of the wing disc using *ptc-Gal4*-driven RNAi. This assay induces robust and reproducible apoptosis in the pouch region, which is entirely dependent on Egr and Grnd (De Vreede et al., 2022). However, we saw no inhibition of apoptosis of *dlg*-depleted cells when PTP10D was co-depleted (Fig. 1A,B,D).

RNAi-mediated transcript depletion can lead to residual levels of protein, and thresholds for functional signaling are seldom known. Yamamoto et al. added *UAS-Dicer2* (*Dcr2*) to their experiments, although the *PTP10D* RNAi construct used is a short hairpin that should not require Dcr2 for processing into siRNAs. When included in the *ptc>dlg-KD* assay, Dcr2 enhanced apoptosis driven by a long-inverted repeat RNA that depletes Dlg, as expected. However, Dcr2 had no impact on the ability of co-depleted PTP10D to reduce apoptosis (Fig. 1C,D). Assessing the efficacy of the *PTP10D* RNAi construct by antibody staining revealed no signal in depleted cells, and there was no enhancement seen when Dcr2 was included (Fig. 1E-H).

The experiments above take place in the wing imaginal disc. To determine whether the contribution of PTP10D was greater in the eye disc than the wing disc, we generated MARCM clones of the *scrib^1^* allele that also express *PTP10D* RNAi under the control of *eyFLP*. The original *scrib^1^ FRT82b PTP10D* RNAi recombinant stock used in Yamamoto et al. has been lost. We obtained the *scrib^1^ FRT82b* stock from which this recombinant was made, and found that in our hands it gave a similar size of eyMARCM clones (5-10%) to that reported by Yamamoto et al. We note that this clone size is smaller than the *scrib^1^ FRT82b* stock used by Liu et al. (2022), which gave typically ∼15% coverage for Liu et al. and ∼25% for our group. For the below experiments we worked primarily with Liu et al.'s *scrib^1^ FRT82b* chromosome and a *scrib^1^ FRT82b PTP10D* RNAi recombinant derived from it.

**Fig. 2.**
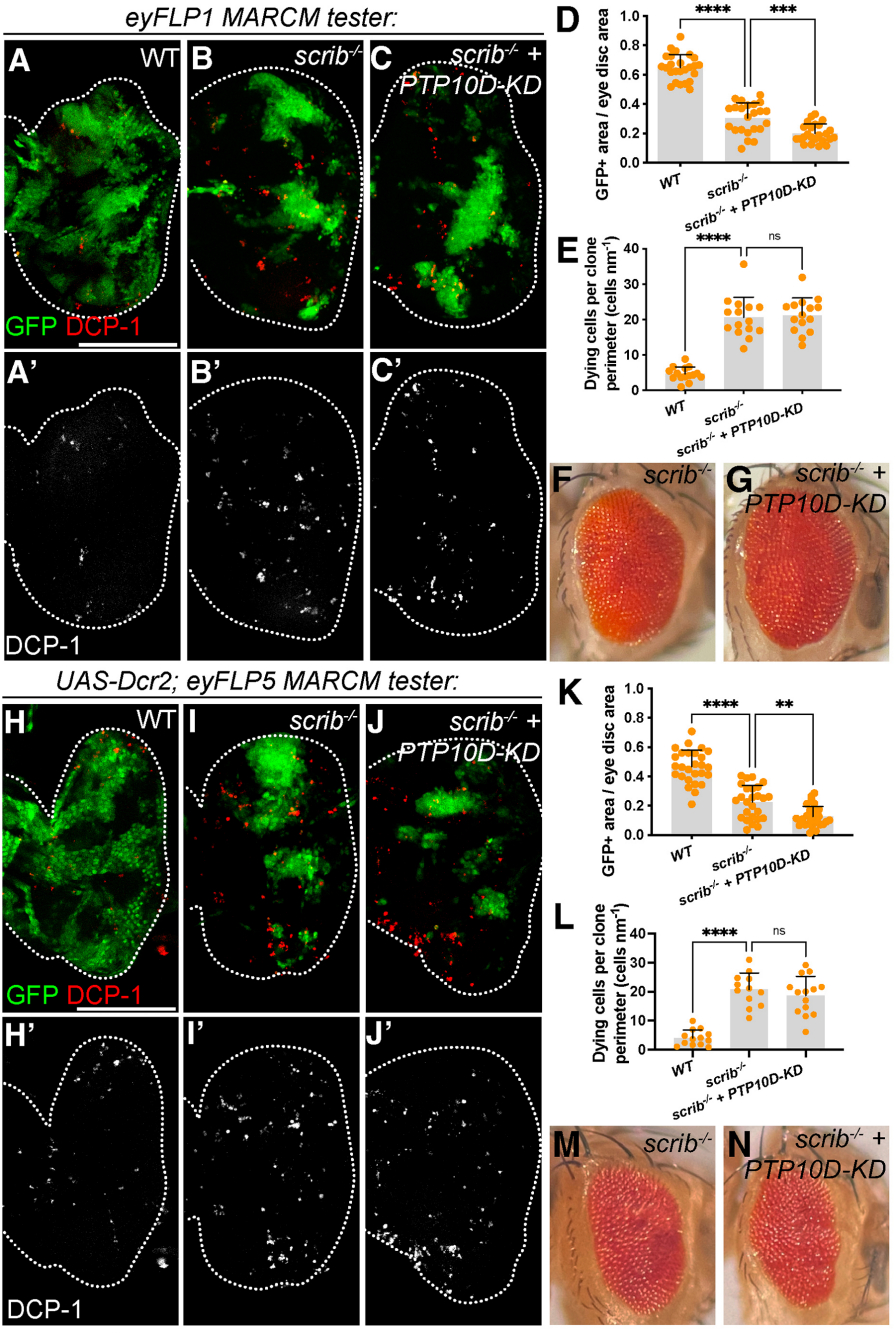
**PTP10D depletion does not rescue *scrib* clone removal.** (A-G) *eyFLP1*-generated *scrib* clones are eliminated from the eye disc (B; control in A) and *PTP10D-KD* does not rescue *scrib* clone elimination (C). Quantitation of clone area in D (mean±s.d., one-way ANOVA test, *n*=23 for WT, *n*=23 for *scrib*, *n*=24 for *scrib+PTP10D-KD*) and apoptosis along the clone boundary in E (mean±s.d., one-way ANOVA test, *n*=15 for WT, *n*=15 for *scrib*, *n*=15 for *scrib+PTP10D-KD*). Adult eyes of *scrib* and *scrib+PTP10D-KD* flies show a rough eye phenotype that is not enhanced with PTP10D depletion (F,G). (H-N) Additional expression of Dcr2 in *eyFLP5*-generated *scrib* clones shows clone elimination (I; control in H) that is not enhanced by *PTP10D-KD* (J). Quantitation of clone area in K (mean±s.d., one-way ANOVA test, *n*=27 for WT, *n*=26 for *scrib*, *n*=29 for *scrib+PTP10D-KD*) and apoptosis along the clone boundary in L (mean±s.d., one-way ANOVA test, *n*=13 for WT, *n*=12 for *scrib*, *n*=14 for *scrib+PTP10D-KD*). Adult eyes of *scrib* and *scrib+PTP10D-KD* flies show similar rough eye phenotypes (M,N). Scale bars: 100 µm in A and H. Statistical significance is indicated with *P≤0.05, **P≤0.01, ***P≤0.001, and ****P≤0.0001.

In agreement with previous work, *scrib^1^* eyMARCM clones were significantly smaller than control clones. However, no increase in clone size was seen when PTP10D was co-depleted; in many experiments a small but significant decrease was seen in the double-depleted clones (Fig. 2A-D). *scrib^1^* clones showed PTP10D mislocalization and co-depletion of PTP10D efficiently ablated the protein from clones, while another apically polarized protein, Grnd, was equally mislocalized in *scrib^1^* clones as well as clones that co-depleted PTP10D (Fig. S1A-D). Measurements of apoptotic cells along the clone periphery also failed to show a difference (Fig. 2E). This result was the same when the *eyFLP1* insertion was used alone, or when the *eyFLP5* insertion was used in conjunction with *UAS-Dcr2* (Fig. 2H-L). Adult flies of both genotypes eclosed with slightly rough eyes resembling those previously reported for *scrib* clones alone (Fig. 2F,G,M,N) (Brumby and Richardson, 2003).

**Fig. 3.**
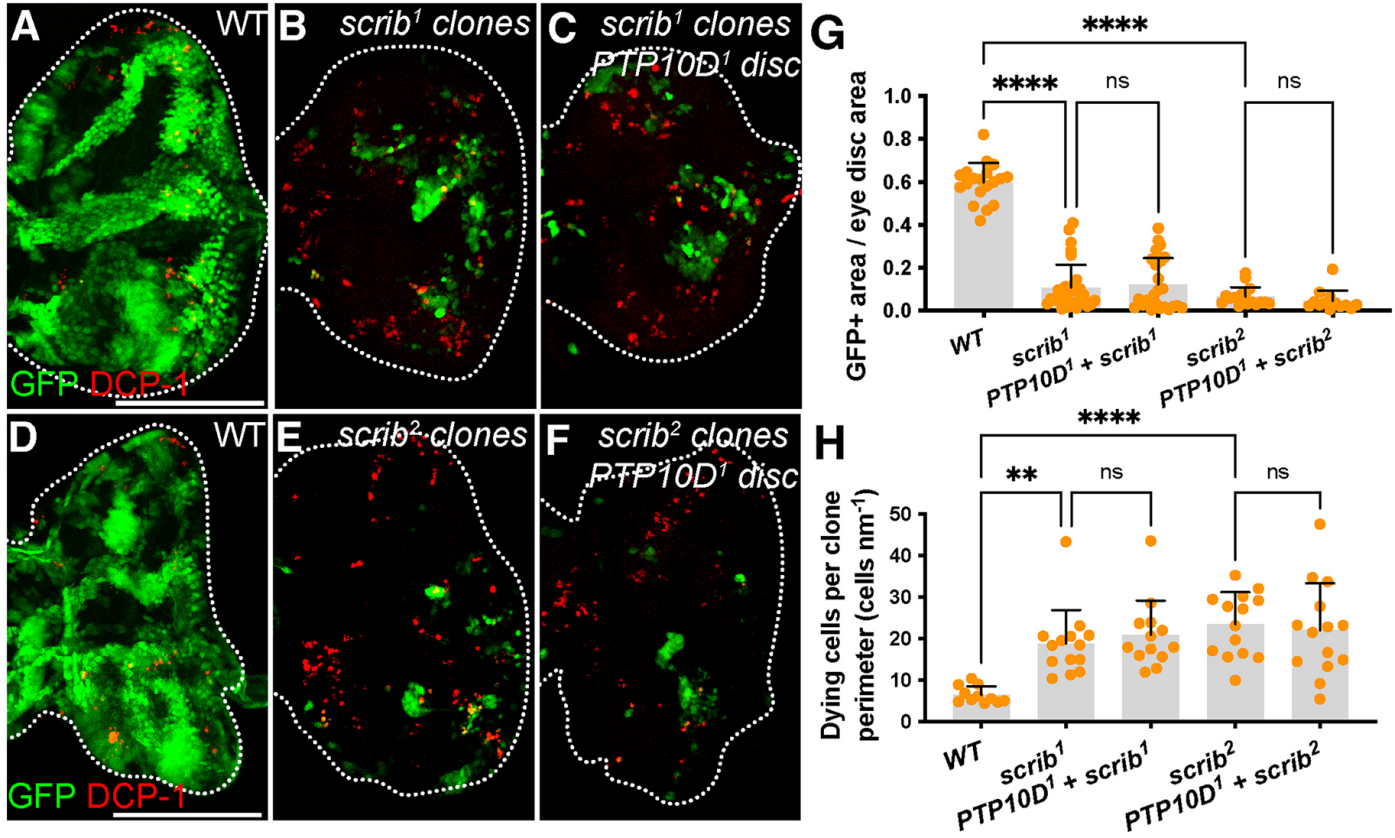
***PTP10D*-null larvae efficiently eliminate *scrib* clones.** (A-H) Animals devoid of *PTP10D* eliminate *scrib^1^* clones as efficiently as animals carrying WT *PTP10D* (B, C; control in A). This is the case for *scrib^2^* clones in animals with WT *PTP10D* or null for *PTP10D* as well (E, F; control in D). Quantitation of clone area in G (mean±s.d., one-way ANOVA test, *n*=19 for WT, *n*=31 for *scrib^1^*, *n*=24 for *PTP10D^1^+scrib^1^*, *n*=17 for *scrib^2^*, *n*=13 for *PTP10D^1^+scrib^2^*) and apoptosis along the clone boundary in H (mean±s.d., one-way ANOVA test, *n*=11 for WT, *n*=14 for *scrib^1^*, *n*=13 for *PTP10D^1^+scrib^1^*, *n*=14 for *scrib^2^*, *n*=14 for *PTP10D^1^+scrib^2^*). Scale bars: 100 µm in A, and D. Statistical significance is indicated with *P≤0.05, **P≤0.01, ***P≤0.001, and ****P≤0.0001.

To rigorously test the requirement for PTP10D in *scrib* cells, we generated mitotic clones in eye discs of larvae hemizygous for a null *PTP10D* allele (Sun et al., 2000). Again, using a *scrib^1^* allele, we found no difference in clone size compared to larvae carrying a WT copy of *PTP10D* (Fig. 3A-C,G). The same result was seen with clones for a second, null *scrib^2^* allele (Fig. 3D-G). In agreement, clones generated with either the *scrib^1^* or *scrib^2^* allele showed no change in apoptosis along the clone boundary in PTP10D WT and null larvae (Fig. 3H). Although we note that all cells in the above experiments lack *PTP10D*, the results fail to support an obligate role for PTP10D in polarity-deficient cell elimination.

We further assessed a role for PTP10D by using other methods to induce heterotypic genotypes of polarity-deficient clones. We used *hsFLP* to induce mitotic MARCM clones of either *dlg* or *lgl* in the wing disc; no difference in size was seen when PTP10D was co-depleted (Fig. S2A-H). The same result was seen with *dlg* clones in the eye disc (Fig. S2I-L). We also used the *FLPout GAL4* system to co-deplete gene products in wing discs. Depletion of either *scrib* or *dlg* caused PTP10D mislocalization and efficient clone elimination, as with mitotic clones of null alleles, but once again co-depletion of PTP10D did not increase clone size (Fig. S2M-U). Thus, extensive experiments failed to detect a role for PTP10D in promoting elimination of polarity-deficient cells.

### Varying husbandry conditions do not reveal a PTP10D role in *scrib* clone elimination

**Fig. 4.**
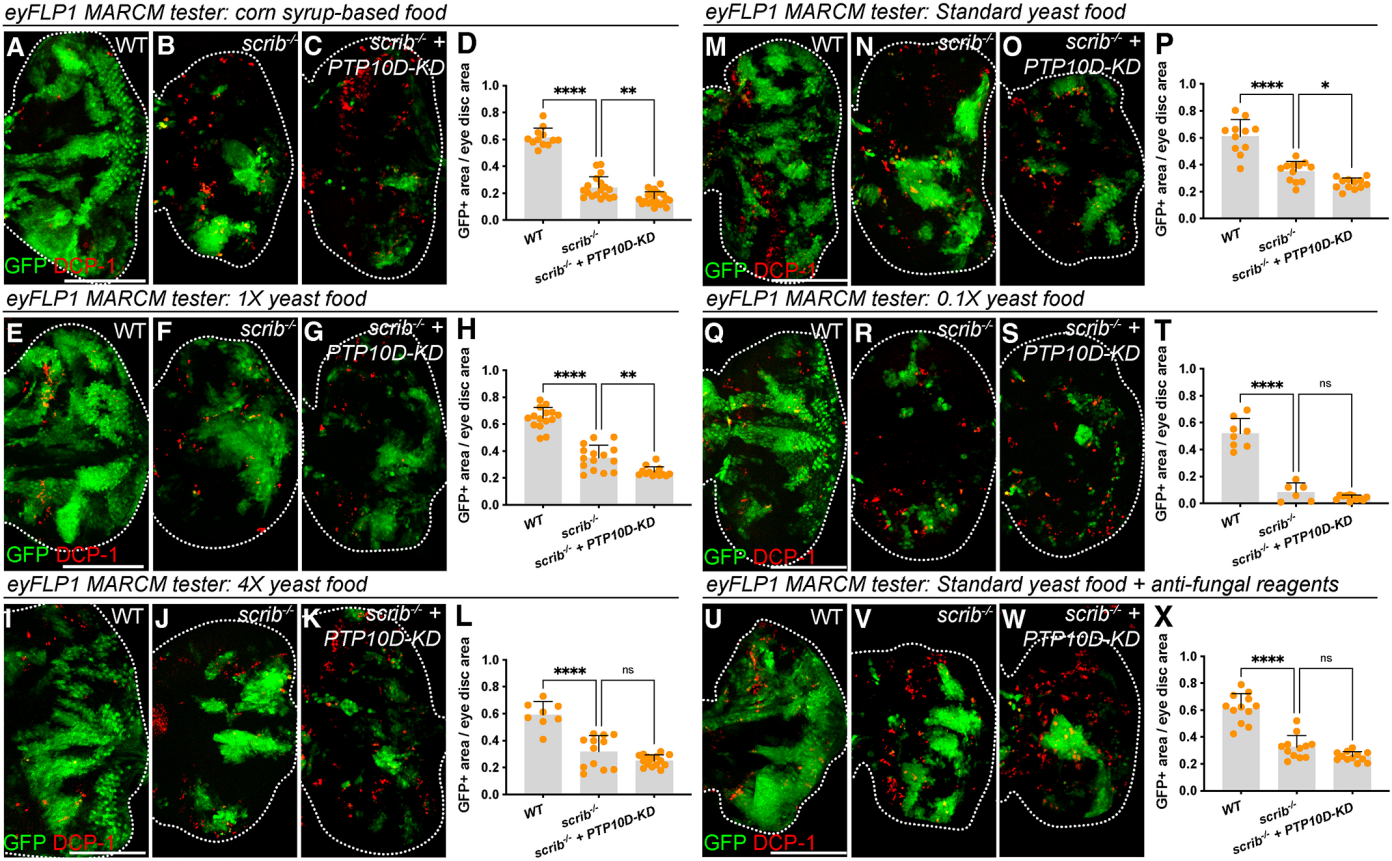
**Varying husbandry conditions do not change outcome of PTP10D depletion.** (A-D) Larvae raised on corn-syrup-based food show efficient elimination of *scrib* clones (B; control in A) and *PTP10D-KD* does not rescue clone elimination (C). Quantitation in D (mean±s.d., one-way ANOVA test, *n*=12 for WT, *n*=18 for *scrib*, *n*=20 for *scrib+PTP10D-KD*). (E-L) Larvae raised on 1X yeast food [E-G; quantitation in H (mean±s.d., one-way ANOVA test, *n*=15 for WT, *n*=15 for *scrib*, *n*=13 for *scrib+PTP10D-KD*)] as well as on 4X yeast food [I-K; quantitation in L (mean±s.d., one-way ANOVA test, *n*=8 for WT, *n*=12 for *scrib*, *n*=15 for *scrib+PTP10D-KD*)] show comparable elimination of *scrib* clones (F,J) and this is not rescued by *PTP10D-KD* (G,K). (M-T) Larvae raised on 0.1X yeast food [Q-S; quantitation in T (mean±s.d., one-way ANOVA test, *n*=8 for WT, *n*=6 for *scrib*, *n*=11 for *scrib+PTP10D-KD*)] show smaller *scrib* clones compared to standard yeast food [M-O; quantitation in P (mean±s.d., one-way ANOVA test, *n*=11 for WT, *n*=12 for *scrib*, *n*=12 for *scrib+PTP10D-KD*)], but *PTP10D-KD* does not rescue *scrib* clone elimination on either food (O,S). (U-X) Larvae raised on standard yeast food supplemented with anti-fungal reagents show efficient elimination of *scrib* clones (V; control in U) and *PTP10D-KD* does not rescue clone elimination (W). Quantitation in X (mean±s.d., one-way ANOVA test, *n*=12 for WT, *n*=12 for *scrib*, *n*=12 for *scrib+PTP10D-KD*). Scale bars: 100 µm in A, E, I, M, Q, and U. Statistical significance is indicated with *P≤0.05, **P≤0.01, ***P≤0.001, and ****P≤0.0001.

To explore what might account for the discrepancy between our experiments and those of Yamamoto et al. and Liu et al., we considered husbandry conditions. In all cases flies were raised at 25°C, but food conditions are known to differ widely among *Drosophila* labs, even those that are considered ‘standard diets’ (Lesperance and Broderick, 2020). Moreover, Agrawal et al. showed that a low protein diet can increase circulating Egr levels and reduce circulating dILP levels, while Sanaki et al. found that heightened insulin levels can increase *scrib* clone survival (Agrawal et al., 2016; Sanaki et al., 2020). We considered whether different protein levels might account for the different results. Our laboratory food follows a common molasses-based recipe similar to that used by the Janelia Research Center among others (Table S1), so we repeated *eyFLP MARCM* experiments on food following three other different recipes: (1) corn syrup-based food used by the Bloomington *Drosophila* stock center, (2) the ‘1X yeast’ recipe of Sanaki et al., and (3) the ‘4X yeast’ recipe of Sanaki et al., which has been shown to increase circulating dILP levels (Sanaki et al., 2020). However, no increase in *scrib* clone size was seen when PTP10D was co-depleted on any of these three food sources (Fig. 4A-L).

We then compared two further recipes: the standard food recipe of Sanaki et al. and a ‘0.1X yeast’ recipe that contains 10% of the yeast in the standard food. We assayed fat bodies carrying a GFP-tagged Pleckstrin Homology domain (tGPH) that binds to plasma membrane PIP3 and is used to compare insulin pathway activity levels (Britton et al., 2002). tGPH measurements confirmed that larvae raised on 0.1X yeast had lower insulin, signaling than Sanaki et al. standard food (Fig. S3A-C). Although *scrib* clone size was significantly decreased on the 0.1X yeast food compared to Sanaki et al. standard food, no difference was seen when PTP10D was co-depleted in *scrib* clones from larvae raised on 0.1X yeast or Sanaki et al. standard food (Fig. 4M-T). No difference was also seen when PTP10D was depleted in *scrib* clones raised on Sanaki et al. standard food supplemented with or without anti-fungal and preservative reagents (Fig. 4U-X). By comparison, a nearly complete rescue of apoptosis in the polarity-deficient cells generated using the *ptc>dlg-KD* assay was seen when Grnd was co-depleted in cultures raised on either 0.1X or 4X food (Fig. S3D-S), as previously described on molasses food (De Vreede et al., 2022). These results suggest that the requirement for Egr-Grnd signaling is significantly more robust to food and other conditions than the requirement for PTP10D.

**Fig. 5.**
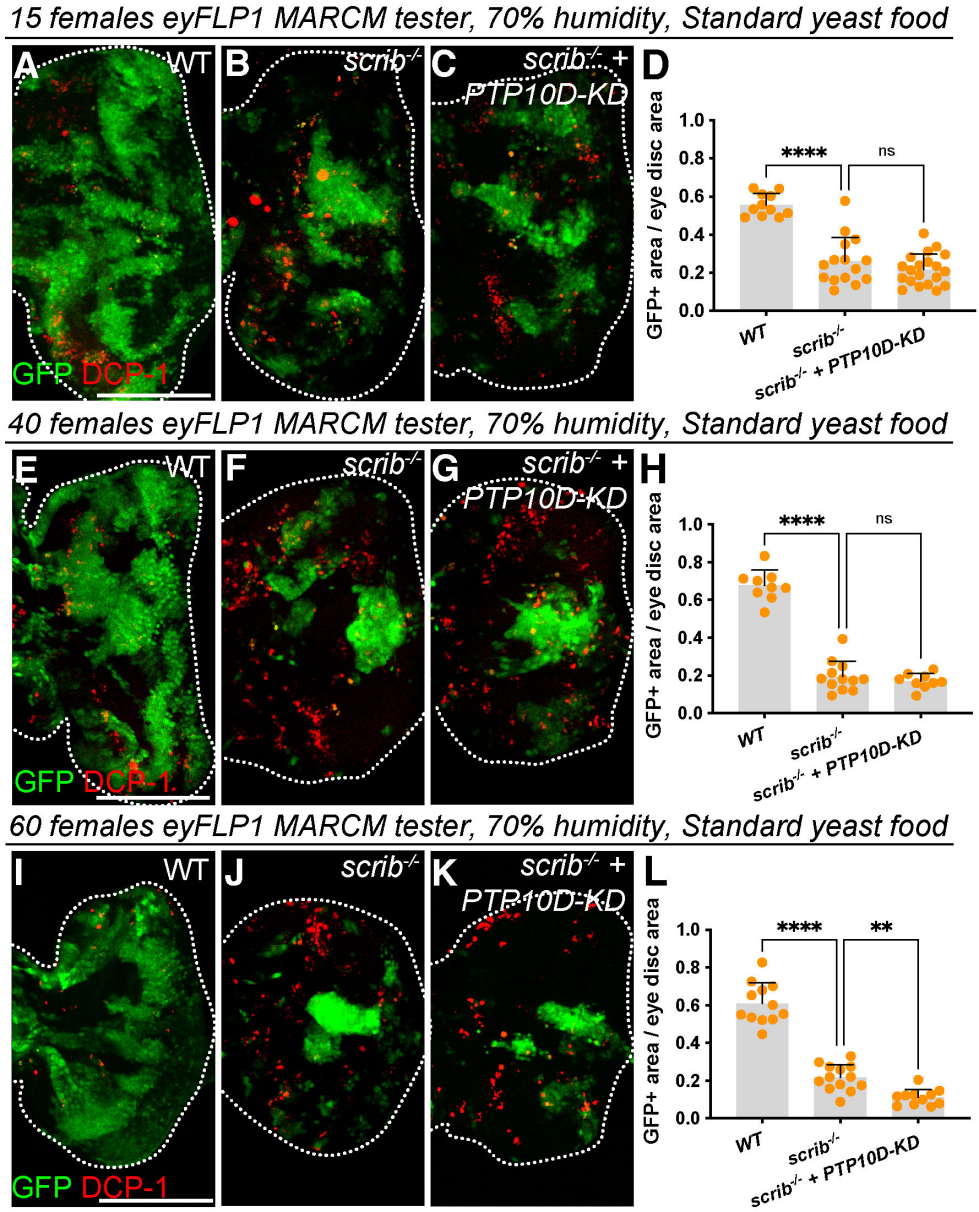
**Varying crowding conditions do not change outcome of PTP10D depletion.** This set of experiments used standard yeast food to raise larvae and was carried out in an independent incubator set to 25°C, 70% humidity control and 12 h light/dark cycles. (A-L) Crosses raised in wide vials under medium larval density (egg laying of 15 females) show removal of *scrib* clones that is not changed by *PTP10D-KD* [A-C; quantitation in D (mean±s.d., one-way ANOVA test, *n*=11 for WT, *n*=15 for *scrib*, *n*=20 for *scrib+PTP10D-KD*)]. Larvae raised in narrow vials under high-density conditions [E-G; quantitation in H (mean±s.d., one-way ANOVA test, *n*=9 for WT, *n*=12 for *scrib*, *n*=9 for *scrib+PTP10D-KD*); egg laying of 40 females] as well as very high-density conditions [I-K; quantitation in L (mean±s.d., one-way ANOVA test, *n*=12 for WT, *n*=13 for *scrib*, *n*=11 for *scrib+PTP10D-KD*); egg laying of 60 females] show removal of *scrib* clones (F,J) that is not rescued by *PTP10D-KD* (G,K). Scale bars: 100 µm in A, E, and I. Statistical significance is indicated with *P≤0.05, **P≤0.01, ***P≤0.001, and ****P≤0.0001.

It is known that larval crowding can influence food availability (Klepsatel et al., 2018). We tested cultures on Sanaki et al. standard food under different density conditions in both wider (∼29 mm) vials and narrower (∼25 mm) vials. No difference was seen between the size of *scrib* clones with or without PTP10D depletion in these cases (Fig. 5A-L). No difference was also seen when medium density cultures on Sanaki et al. standard food were raised in a different humidified, light controlled incubator. Thus, we have been unable to find a culture parameter in which, in our hands, PTP10D depletion can rescue the size of *scrib* mutant clones.

We conclude that, under a range of frequently used conditions, PTP10D within polarity-deficient cells is not an obligate requirement for their elimination in a heterotypic cellular context. This conclusion is based on tests using different polarity-deficient mutants, imaginal tissues, induction protocols, food recipes and husbandry conditions. Importantly, many of these assays were carried out with identical stocks provided by another group who are indeed able to detect an increase in *scrib* clone representation when PTP10D is co-depleted. We have not extensively investigated the influence of the proposed PTP10D partner Sas in WT cells on *scrib* cell elimination, but on molasses food we saw no difference in clone size nor border apoptosis, although eye defects resembling those shown by Yamamoto et al. were reliably obtained (Fig. S4A-H). Despite varying many parameters, we are unable to offer an explanation for the discrepancy with published results from other labs. Nevertheless, we feel that it is reasonable to suggest that the modulating impact of PTP10D on Egr-dependent polarity-deficient cell elimination may depend on different culture situations.

## MATERIALS AND METHODS

### *Drosophila* genetics

*w1118* larvae were used as control. Experiments using temperature-sensitive *ptc-Gal4*, were transferred to 29°C 60 h±12 h after egg laying (AEL) and dissected after additional 72 h at 29°C, plus an additional 24 h at 29°C for crosses on 0.1X yeast food. *eyFLP*-induced eye imaginal disc clones were dissected at 120 h±24 h AEL, plus an additional 24 h for crosses on 0.1X yeast food. *hsFLP* wing and eye imaginal disc clones were induced by a 15-min heat shock 48 h±12 h AEL. Larvae carrying *dlg-KD* clones were raised on 18°C and transferred to 29°C 24 h before dissection. For *scrib-KD* clones, as well as *dlg* or *lgl* mutant clones, larvae were raised on 25°C after heat shock. Wandering L3 larvae were dissected for all experiments. The following fly stocks were used: *w1118* #5905, *ptc-Gal4* #2017, *tub-Gal80-ts* #7019, *UAS-PTP10D-RNAi* #39001, *UAS-scrib-RNAi* #39073, *UAS-dlg-RNAi (II)* #39035, *UAS-Dicer2* #24650, *tGPH* #8164, *PTP10D^1^* #5810, *Act>CD2>Gal4 UAS-RFP* #30558, and *hsFLP* #8862 were obtained from the Bloomington *Drosophila* Stock Center. *UAS-dlg-RNAi (III)* #41136, and *UAS-grnd-RNAi* #104538 are from the Vienna *Drosophila* Resource Center. Other *Drosophila* strains used were: *eyFLP1; Act>y+>Gal4, UAS-GFP; FRT82b, tub-Gal80* (Pagliarini and Xu, 2003), *eyFLP1; Act>y+>Gal4, UAS-GFP; FRT82b, sas^eld−4^, tub-Gal80* and *UAS-Dicer2; eyFLP5, Act>y+>Gal4, UAS-GFP; FRT82b, tub-Gal80* (Yamamoto et al., 2017), *scrib^1^ FRT82b* and *scrib^2^ FRT82b* (Bilder and Perrimon, 2000)*, scrib^1^, PTP10D-RNAi, FRT82b* (Liu et al., 2022), *dlg^m52^ FRT19a* (Perrimon, 1988), *lgl^27S3^ FRT40a* (Grzeschik et al., 2007), isogenized *FRT19a, FRT40a, FRT82b, as well as hsFLP, FRT19a, tub-Gal80; Act-Gal4, UAS-GFP and UAS-GFP, hsFLP; tub-Gal80, FRT40a; tub-Gal4. Drosophila* strains are listed in Table S2, and detailed genotypes are indicated in Table S3.

### Husbandry conditions and food recipes

Experimental crosses were raised at 25°C on molasses-based food in medium density conditions in wider fly vials (29.21 mm diameter), unless otherwise indicated. For high and very high-density conditions in Fig. 5, eggs collected from separate crosses with 20 virgins were merged into one narrow (25 mm) vial, which limits nutrient availability and access to surface air while increasing stressors such as exposure to wastes. This approach yielded average numbers of 230, 600, and 900 L3 larvae in medium, high and very high-density conditions, respectively. These conditions are numerically and visually comparable to those depicted in (Henry et al., 2018) when vial differences are taken into account. Detailed information of nutritional ingredients per food can be found in Table S1. Molasses-based food was prepared from single ingredients while corn syrup-based food was prepared from Nutri-Fly Bloomington formulation packets, both in quantities of 10-15 L. Standard food, 0.1X, 1X and 4X yeast food based on Sanaki et al. recipes was prepared freshly in quantities of 200 ml by dissolving the ingredients for 6 min in a microwave oven. Anti-fungal reagents included in Fig. 4U-X were 15 ml 10% Tegosept dissolved in ethanol and 5 ml propionic acid per 1 L of food. For experiments in Figs 4, 5, and Fig. S3, crosses laid eggs and were raised directly on the indicated food until dissection. The 0.1× yeast food caused a ∼1-day delay until the animals developed into wandering L3 larvae.

### Immunohistochemistry and microscopy

Imaginal eye and wing discs as well as larval fat bodies were dissected, fixed in 4% paraformaldehyde for 20 min, and incubated with the following primary antibodies using standard immunohistochemistry procedures: rabbit anti-DCP-1 (1:100; Cell Signaling Technology, #9578), mouse anti-PTP10D (1:100, DSHB, #8B22f5), and mouse anti-Grnd (1:200, #7D9; De Vreede et al., 2018). Secondary fluorophore-conjugated antibodies were used 1:200 and DNA was visualized with DAPI, used 1:1000. Antibodies are listed in Table S2. Micrographs were taken on a Zeiss LSM700 confocal and processed with ImageJ as well as Adobe Photoshop CC. Data were collected as 16-bit per channel.

### Quantifications and statistics

To determine central DCP-1 enrichment in experiments using *ptc-Gal4*, the mean gray value of the middle third region of the wing pouch was measured and divided by that of the outer two-thirds of the wing pouch after deducting the background signal from both values. PTP10D fluorescence was determined by measuring the mean gray of the middle third region and divided by that of the outer two-thirds. Clone size in eye and wing imaginal discs was determined by measuring the fluorescently labeled area and divided by that of the entire area of the eye disc or wing pouch. Apoptosis along the clone boundary was quantified by counting DCP-1-positive cells that were marked by GFP within 10 µm of the clone boundary, accounting to about 2-3 cells within proximity of WT cells. To assess insulin signaling in larval fat bodies, tGPH mean gray values were measured at the cell membrane as well as in the cytosol of fat body cells and divided. Each data point for measurements of central DCP-1 enrichment, PTP10D fluorescence, GFP+ or RFP+ area per eye disc area, and dying cells per clone perimeter represents one imaginal disc. Each data point for tGPH measurements represents one fat body cell. Scatter dot-plots show the mean as grey columns and error bars indicating standard deviation. Statistical analysis was performed with Microsoft Excel and GraphPad Prism 9. Two-sample comparisons used the unpaired *t*-test and multiple sample comparisons used the ordinary one-way ANOVA test to determine significance.

## Supplementary Material

10.1242/biolopen.059525_sup1 Supplementary information
