# Supplementary material for: PTP10D-mediated cell competition is not obligately required for elimination of polarity-deficient clones
Source: Biol Open. 2022 Nov 10;11(11):bio059525. doi: 10.1242/bio.059525 (PMC9672856; doi:10.1242/bio.059525)
Supplement: Supplementary information [file biolopen-11-059525-s1.pdf]

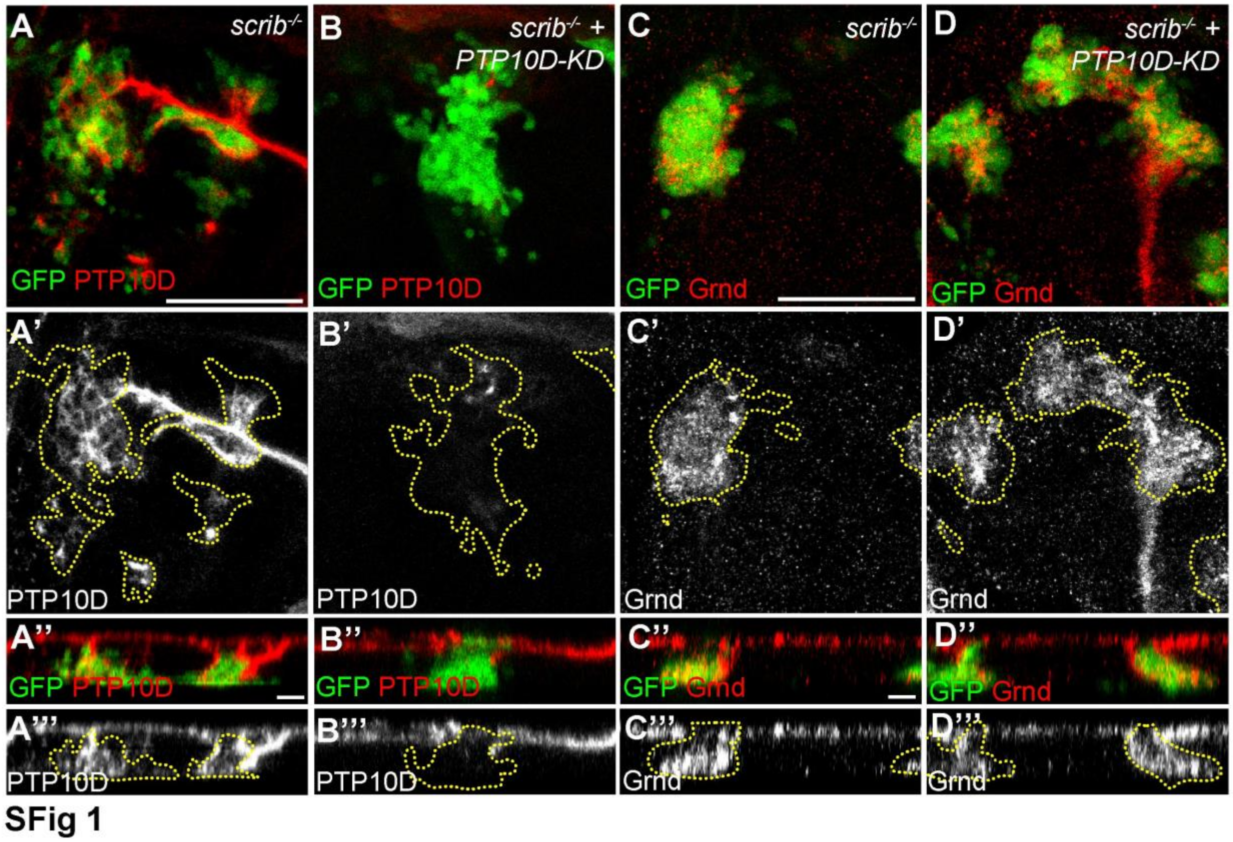

Fig. S1. PTP10D depletion efficiently ablates PTP10D protein from *scrib* clones

(A-B): *scrib* clones in the eye disc show mislocalization of PTP10D (A), while *scrib* clones expressing *PTP10D-KD* are depleted of PTP10D protein (B). A'', A''' as well as B'', B''' show X-Z cross sections of A and B respectively.

(C-D): *scrib* clones (C) as well as *scrib* clones expressing *PTP10D-KD* (D) in the eye disc show mislocalization of Grnd. C'', C''' as well as D'', D''' show X-Z cross sections of C and D respectively. Scale bars 50 µm in A, and C, 10 µm in A'', and C''.

Biology Open • Supplementary information

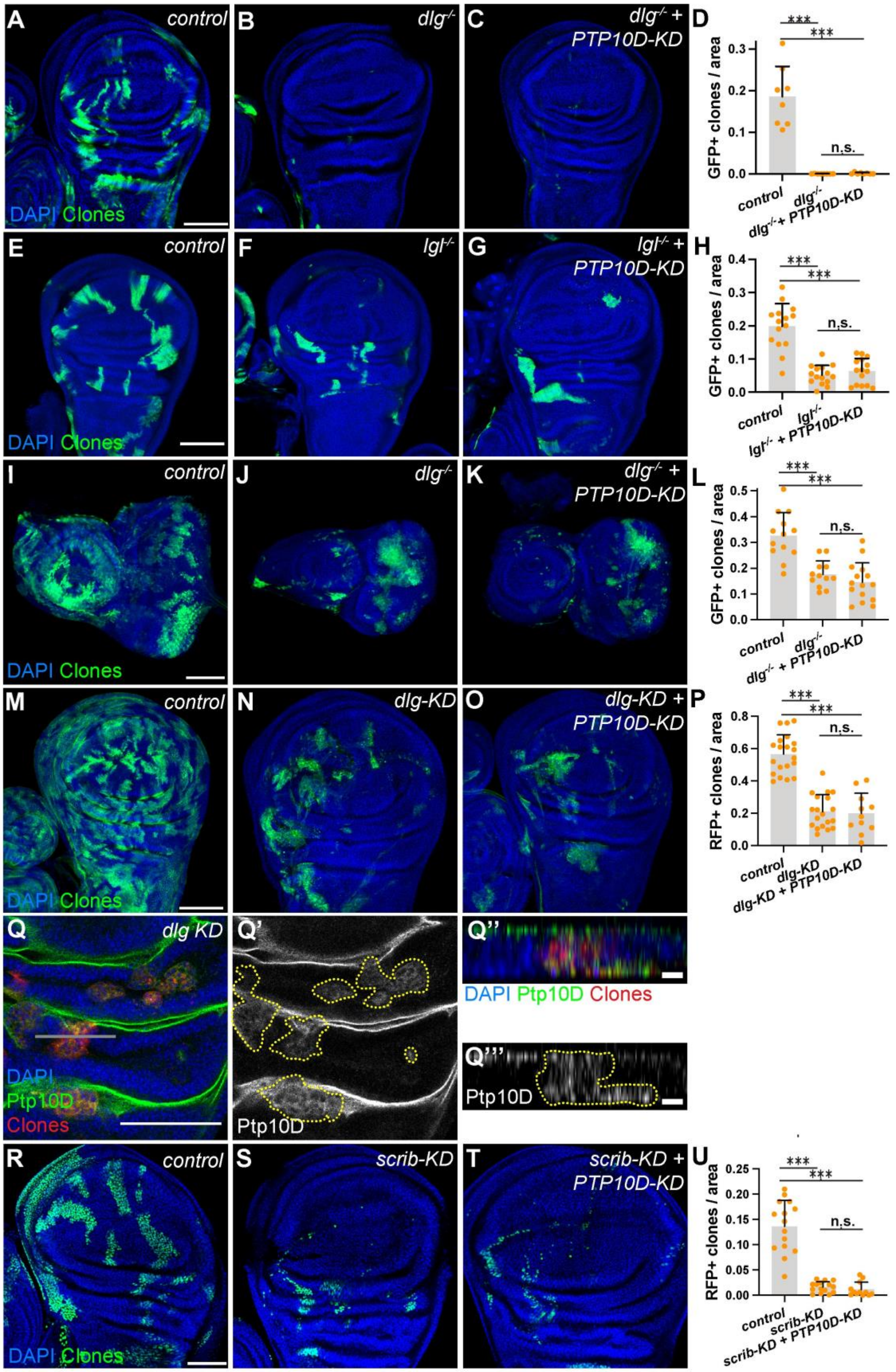

SFig 2

Biology Open • Supplementary information

Fig. S2. Co-depletion of *scrib* class genes and PTP10D

(A-D): *dlg* mutant clones in the wing disc undergo cell elimination (B; control in A) even when PTP10D is co-depleted (C). Quantitation in D (mean±s.d., one-way ANOVA test, n=8 for control, n=8 for *dlg*, n=8 for *dlg+PTP10D-KD*).

(E-H): *lgl* mutant clones in the wing disc undergo cell elimination (F; control in E) even when PTP10D is co-depleted (G). Quantitation in H (mean±s.d., one-way ANOVA test, n=15 for control, n=15 for *lgl*, n=15 for *lgl+PTP10D-KD*).

(I-L): *dlg* mutant clones in the eye disc undergo cell elimination (J; control in I) even when PTP10D is co-depleted (K). Quantitated in L (mean±s.d., one-way ANOVA test, n=13 for control, n=12 for *dlg*, n=15 for *dlg+PTP10D-KD*).

(M-Q): Dlg-depleted clones in the wing disc undergo cell elimination (N; control in M) even when PTP10D is co-depleted (O). Quantitation in P (mean±s.d., one-way ANOVA test, n=20 for control, n=19 for *dlg-KD*, n=11 for *dlg-KD+PTP10D-KD*). *dlg-KD* clones in the wing disc show PTP10D mislocalization (Q). Q'' shows a X-Z cross section of magnified area of Q (marked in Q by grey line).

(R-U): Scrib-depleted clones in the wing disc undergo cell elimination (S; control in R) even when PTP10D is co-depleted (T). Quantitation in U (mean±s.d., one-way ANOVA test, n=15 for control, n=15 for *scrib-KD*, n=12 for *scrib-KD+PTP10D-KD*).

All wing discs are oriented with their ventral side up and anterior side left. Scale bars 100 µm in A, E, I, M, and R, 50 µm in Q, 10 µm in Q'', and Q'''. Statistical significance is indicated with * p≤0.05, **p≤0.01, ***p≤0.001, and **** p≤0.0001.

Biology Open • Supplementary information

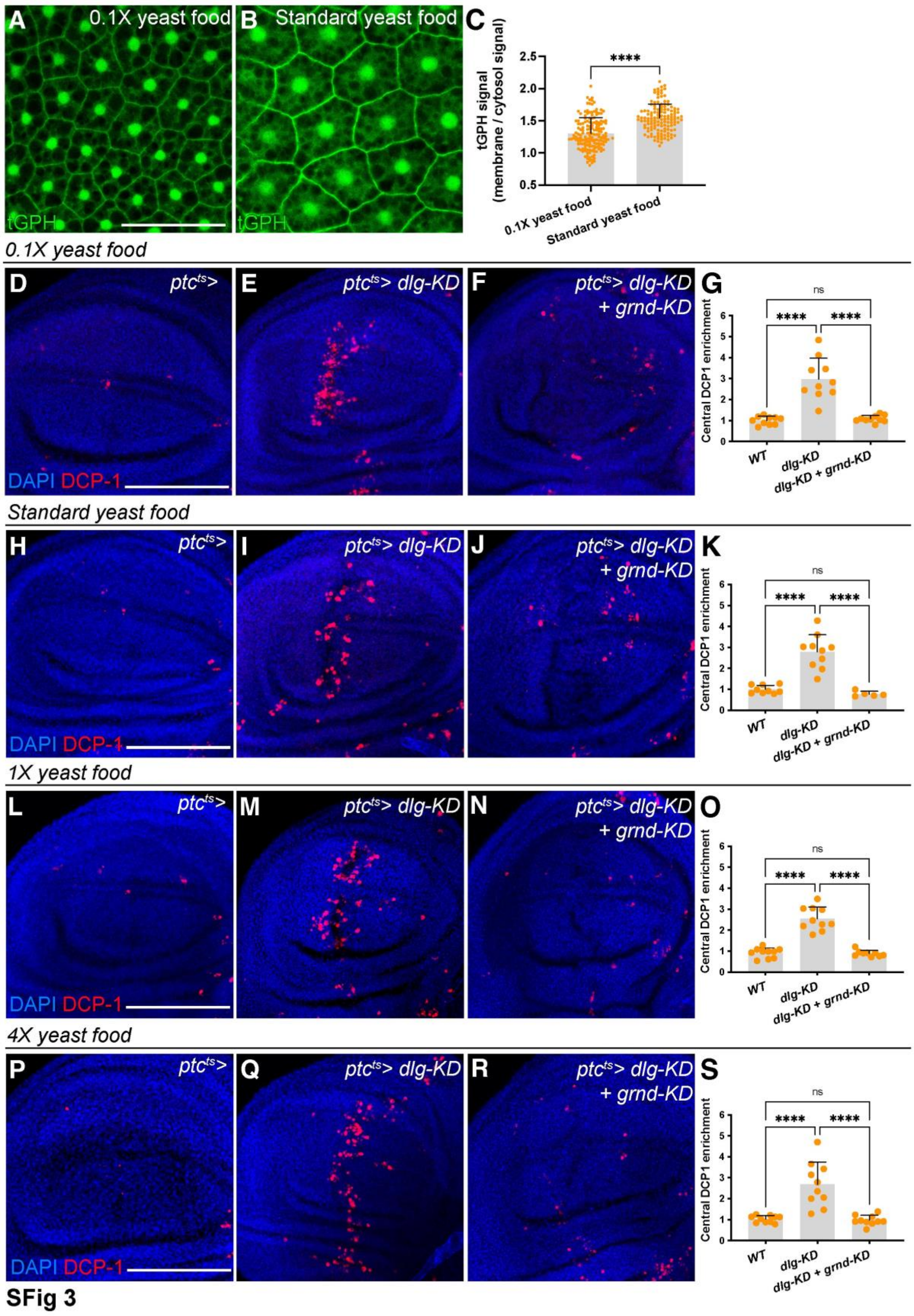
A
0.1X yeast food
tGPH
B
Standard yeast food
tGPH
C
tGPH signal (membrane / cytosol signal)
****
0.1X yeast food
Standard yeast food
0.1X yeast food
D
ptc^ts>
E
ptc^ts> dlg-KD
F
ptc^ts> dlg-KD + grnd-KD
DAPI DCP-1
G
Central DCP1 enrichment
ns
****
****
WT
dlg-KD
dlg-KD + grnd-KD
Standard yeast food
H
ptc^ts>
I
ptc^ts> dlg-KD
J
ptc^ts> dlg-KD + grnd-KD
DAPI DCP-1
K
1X yeast food
L
ptc^ts>
M
ptc^ts> dlg-KD
N
ptc^ts> dlg-KD + grnd-KD
DAPI DCP-1
O
4X yeast food
P
ptc^ts>
Q
ptc^ts> dlg-KD
R
ptc^ts> dlg-KD + grnd-KD
DAPI DCP-1
S

SFig 3

Fig. S3. Polarity-deficient cell elimination is entirely dependent on Grnd

(A-C): Insulin signaling, assayed by tGPH (green, comparison of plasma membrane and cytosolic levels) is reduced on low protein 0.1X yeast food (A) compared to standard food following the recipe of Sanaki et al. (B). Quantitated in C (mean±s.d., unpaired t-test, n=160 for 0.1X yeast food, n=130 for Standard yeast food).

(D-S): Polarity-deficient cells, generated through *ptc-Gal4*-driven *dlg* depletion, induce apoptosis along the A-P boundary on low protein 0.1X yeast food (E; control in D), standard food (I; control in H), 1X yeast food (M; control in L), and high protein 4X yeast food (Q; control in P). Elimination of polarity-deficient cells is entirely dependent on Grnd and *grnd* depletion inhibits apoptosis along the A-P boundary on tested foods (F, J, N, R). Quantitated in G (mean±s.d., one-way ANOVA test, n=10 for WT, n=10 for *dlg-KD*, n=10 for *dlg-KD+grnd-KD*), K (mean±s.d., one-way ANOVA test, n=9 for WT, n=10 for *dlg-KD*, n=5 for *dlg-KD+grnd-KD*), O (mean±s.d., one-way ANOVA test, n=10 for WT, n=10 for *dlg-KD*, n=9 for *dlg-KD+grnd-KD*), S (mean±s.d., one-way ANOVA test, n=10 for WT, n=10 for *dlg-KD*, n=10 for *dlg-KD+grnd-KD*).

Scale bars 100 μm in A, D, H, L, and P. Statistical significance is indicated with *p≤0.05, **p≤0.01, ***p≤0.001, and **** p≤0.0001.

Biology Open • Supplementary information

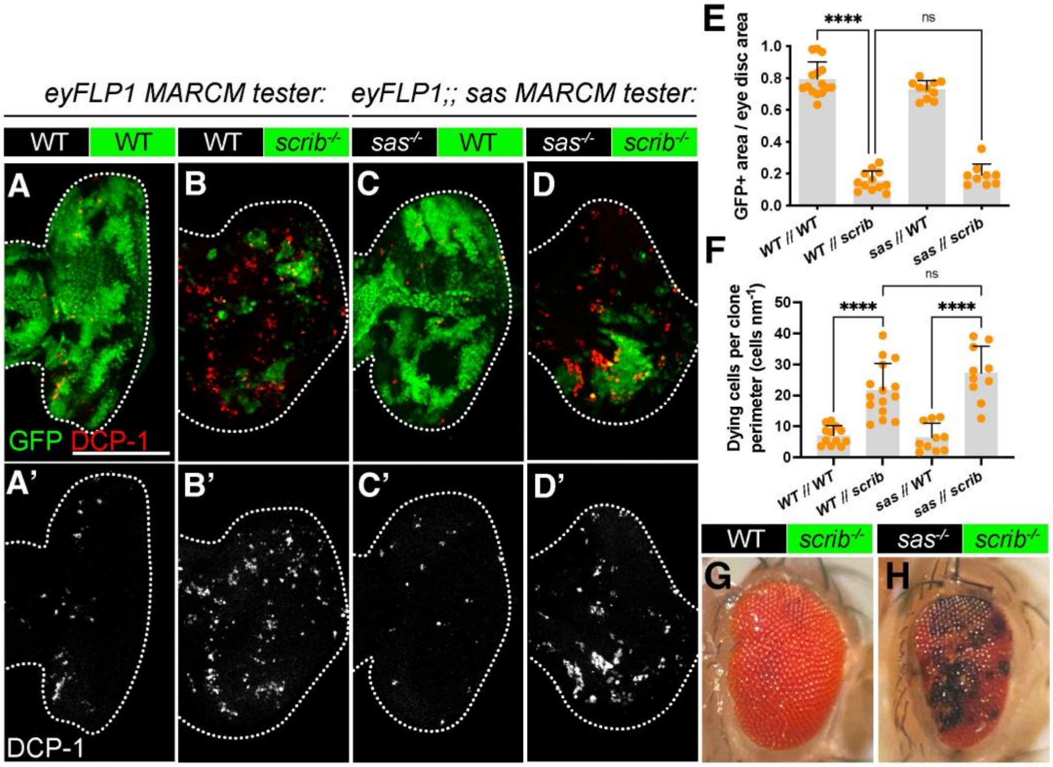

SFig 4

Fig. S4. Minor effect on *scrib* clones by *sas* depletion in neighboring cells

(A-H): *eyFLP1*-generated *scrib* clones are eliminated from the eye disc (B; control in A) and this is not changed when *scrib* clones are surrounded by *sas*$^{eld\text{-}4}$ null mutant cells (D; control in C). Quantitation of clone area in E (mean±s.d., one-way ANOVA test, n=15 for WT//WT, n=12 for WT//*scrib*, n=10 for *sas*//WT*, n=9 for sas*//*scrib*) and apoptosis along the clone boundary in F (mean±s.d., one-way ANOVA test, n=12 for WT//WT, n=15 for WT//*scrib*, n=10 for *sas*//WT, n=10 for *sas*//*scrib*); '//' separates different genotypes of neighboring cells and clones. Adult eyes of WT//*scrib* flies show the typical rough eye phenotype (G). *sas*//*scrib* flies show melanized areas of the adult eye (H).

Scale bars 100 µm in A. Statistical significance is indicated with *p≤0.05, **p≤0.01, ***p≤0.001, and **** p≤0.0001.

Table S1. Fly food Information

**Ingredient**	**Molasses-based food**	**Corn syrup-based food**
Yeast	20.08 g	15.88 g
Soy flour	----	9.18 g
Cornmeal	74.15 g	67.06 g
Molasses	91.45 mL	----
Corn syrup	----	70.59 mL
Agar	6.96 g	5.29 g
10% Tegosept in Ethanol	15.87 mL	10 mL
Propionic Acid	5.25 mL	4.8 mL
Water	Adjusted to total volume after ingredients were added	
Total Volume	1000 mL	1000 mL

**Ingredient**	**Standard food**	**1X yeast food**	**4X yeast food**	**0.1X yeast food**	**Standard food + anti-fungal reagents**
Yeast	40 g	40 g	160 g	4 g	40 g
Cornmeal	40 g	40 g	40 g	40 g	40 g
Dextrose	16 g	----	----	16 g	16 g
Agar	8 g	8 g	8 g	8 g	8 g
10% Tegosept in Ethanol	----	----	----	----	15 mL
Propionic Acid	----	----	----	----	5 mL
Water	Adjusted to total volume after ingredients were added				
Total Volume	1000 mL	1000 mL	1000 mL	1000 mL	1000 mL

Biology Open • Supplementary information

Table S2. Key Resources

Drosophila strains	Source	Reference
w1118	BDSC	BDSC #5905
patched-Gal4	BDSC	BDSC #2017
tubulin-Gal80-ts	BDSC	BDSC #7019
UAS-PTP10D-RNAi (III)	BDSC	BDSC #39001
UAS-scrib-RNAi (II)	BDSC	BDSC #39073
UAS-dlg-RNAI (II)	BDSC	BDSC #39035
UAS-Dicer2 (II)	BDSC	BDSC #24650
tGPH (III)	BDSC	BDSC #8164
PTP10D^{1}	BDSC	BDSC #5810
Act>CD2>Gal4, UAS-RFP (III)	BDSC	BDSC #30558
hsFLP	BDSC	BDSC #8862
UAS-dlg-RNAi (III)	VDRC	VDRC #41136
UAS-grnd-RNAi (II)	VDRC	VDRC #104538
eyFLP1; Act>y+>Gal4, UAS-GFP; FRT82b, tubulin-Gal80	Igaki lab	(Pagliarini & Xu, 2003)
eyFLP1; Act>y+>Gal4, UAS-GFP; FRT82b, sas-eld-4, tubulin-Gal80	Igaki lab	(Yamamoto et al., 2017)
UAS-Dicer2; eyFLP5, Act>y+>Gal4, UAS-GFP; FRT82b, tubulin-Gal80	Igaki lab	(Yamamoto et al., 2017)
scrib^{1}*, FRT82b*	Samakovlis lab in Fig. 2, 4, 5, Fig. S1; Igaki lab in Fig. 3, Fig. S4	(Bilder & Perrimon, 2000)
scrib^{1}*, PTP10D-RNAi, FRT82b*	Samakovlis lab	(Liu et al., 2022)
scrib^{2}*, FRT82b*	Bilder lab	(Bilder & Perrimon, 2000)
dlg^{m52}*, FRT19a*	Perrimon lab	(Perrimon, 1988)
lgl^{27S3}*, FRT40a*	Richardson lab	(Grzeschik et al., 2007)
FRT19a	Bilder lab	
FRT40a	Bilder lab	
FRT82b	Bilder lab	
hsFLP, FRT19a, tub-Gal80; Act-Gal4, UAS-GFP	Bilder lab	

UAS-GFP, hsFLP; tub-Gal80, FRT40a; tub-Gal4		Bilder lab
**Antibodies**	**Dilution**	**Reference**
Rabbit anti-DCP-1	1:100	Cell Signaling #9578
Mouse anti-PTP10D	1:100	DSHB #8B22F5
Mouse anti-Grnd	1:200	Bilder lab #7D9; (de Vreede et al., 2018)

Table S3. Detailed Genotypes

Figure	Panel	Genotype
1	A	*ptc-Gal4, tub-Gal80TS/ +; UAS-dlg-RNAi/ +*
	B	*ptc-Gal4, tub-Gal80TS/ +; UAS-dlg-RNAi/ UAS-PTP10D-RNAi*
	C	*ptc-Gal4, tub-Gal80TS/ UAS-Dcr2; UAS-dlg-RNAi/ UAS-PTP10D-RNAi*
	E	*ptc-Gal4, tub-Gal80TS/ +; +/ +*
	F	*ptc-Gal4, tub-Gal80TS/ +; UAS-PTP10D-RNAi/ +*
	G	*ptc-Gal4, tub-Gal80TS/ UAS-Dcr2; UAS-PTP10D-RNAi/ +*
2	A	*eyFLP.1; Act>y+>Gal4, UAS-GFP/ +; FRT82b/ FRT82b, tub-Gal80*
	B	*eyFLP.1; Act>y+>Gal4, UAS-GFP/ +; scrib1, FRT82b/ FRT82b, tub-Gal80*
	C	*eyFLP.1; Act>y+>Gal4, UAS-GFP/ +; scrib1, FRT82b, UAS-PTP10D-RNAi/ FRT82b, tub-Gal80*
	F	*eyFLP.1; Act>y+>Gal4, UAS-GFP/ +; scrib1, FRT82b/ FRT82b, tub-Gal80*
	G	*eyFLP.1; Act>y+>Gal4, UAS-GFP/ +; scrib1, FRT82b, UAS-PTP10D-RNAi/ FRT82b, tub-Gal80*
	H	*UAS-Dcr2; eyFLP.5, Act>y+>Gal4, UAS-GFP/ +; FRT82b/ FRT82b, tub-Gal80*
	I	*UAS-Dcr2; eyFLP.5, Act>y+>Gal4, UAS-GFP/ +; scrib1, FRT82b/ FRT82b, tub-Gal80*
	J	*UAS-Dcr2; eyFLP.5, Act>y+>Gal4, UAS-GFP/ +; scrib1, FRT82b, UAS-PTP10D-RNAi/ FRT82b, tub-Gal80*
	M	*UAS-Dcr2; eyFLP.5, Act>y+>Gal4, UAS-GFP/ +; scrib1, FRT82b/ FRT82b, tub-Gal80*
	N	*UAS-Dcr2; eyFLP.5, Act>y+>Gal4, UAS-GFP/ +; scrib1, FRT82b, UAS-PTP10D-RNAi/ FRT82b, tub-Gal80*
3	A	*+/>; eyFLP.5, Act>y+>Gal4, UAS-GFP/ +; FRT82b/ FRT82b, tub-Gal80*
	B	*+/>; eyFLP.5, Act>y+>Gal4, UAS-GFP/ +; scrib1, FRT82b/ FRT82b, tub-Gal80*
	C	*PTP10D-1/>; eyFLP.5, Act>y+>Gal4, UAS-GFP/ +; scrib1, FRT82b/ FRT82b, tub-Gal80*
	D	*+/>; eyFLP.5, Act>y+>Gal4, UAS-GFP/ +; FRT82b/ FRT82b, tub-Gal80*
	E	*+/>; eyFLP.5, Act>y+>Gal4, UAS-GFP/ +; scrib2, FRT82b/ FRT82b, tub-Gal80*

Biology Open • Supplementary information

	F	*PTP10D-1/>; eyFLP.5, Act>y+>Gal4, UAS-GFP/ +; scrib2, FRT82b/ FRT82b, tub-Gal80*
4	A	*eyFLP.1; Act>y+>Gal4, UAS-GFP/ +; FRT82b/ FRT82b, tub-Gal80*
	B	*eyFLP.1; Act>y+>Gal4, UAS-GFP/ +; scrib1, FRT82b/ FRT82b, tub-Gal80*
	C	*eyFLP.1; Act>y+>Gal4, UAS-GFP/ +; scrib1, FRT82b, UAS-PTP10D-RNAi/ FRT82b, tub-Gal80*
	E	*eyFLP.1; Act>y+>Gal4, UAS-GFP/ +; FRT82b/ FRT82b, tub-Gal80*
	F	*eyFLP.1; Act>y+>Gal4, UAS-GFP/ +; scrib1, FRT82b/ FRT82b, tub-Gal80*
	G	*eyFLP.1; Act>y+>Gal4, UAS-GFP/ +; scrib1, FRT82b, UAS-PTP10D-RNAi/ FRT82b, tub-Gal80*
	I	*eyFLP.1; Act>y+>Gal4, UAS-GFP/ +; FRT82b/ FRT82b, tub-Gal80*
	J	*eyFLP.1; Act>y+>Gal4, UAS-GFP/ +; scrib1, FRT82b/ FRT82b, tub-Gal80*
	K	*eyFLP.1; Act>y+>Gal4, UAS-GFP/ +; scrib1, FRT82b, UAS-PTP10D-RNAi/ FRT82b, tub-Gal80*
	M	*eyFLP.1; Act>y+>Gal4, UAS-GFP/ +; FRT82b/ FRT82b, tub-Gal80*
	N	*eyFLP.1; Act>y+>Gal4, UAS-GFP/ +; scrib1, FRT82b/ FRT82b, tub-Gal80*
	O	*eyFLP.1; Act>y+>Gal4, UAS-GFP/ +; scrib1, FRT82b, UAS-PTP10D-RNAi/ FRT82b, tub-Gal80*
	Q	*eyFLP.1; Act>y+>Gal4, UAS-GFP/ +; FRT82b/ FRT82b, tub-Gal80*
	R	*eyFLP.1; Act>y+>Gal4, UAS-GFP/ +; scrib1, FRT82b/ FRT82b, tub-Gal80*
	S	*eyFLP.1; Act>y+>Gal4, UAS-GFP/ +; scrib1, FRT82b, UAS-PTP10D-RNAi/ FRT82b, tub-Gal80*
	U	*eyFLP.1; Act>y+>Gal4, UAS-GFP/ +; FRT82b/ FRT82b, tub-Gal80*
	V	*eyFLP.1; Act>y+>Gal4, UAS-GFP/ +; scrib1, FRT82b/ FRT82b, tub-Gal80*
	W	*eyFLP.1; Act>y+>Gal4, UAS-GFP/ +; scrib1, FRT82b, UAS-PTP10D-RNAi/ FRT82b, tub-Gal80*
5	A	*eyFLP.1; Act>y+>Gal4, UAS-GFP/ +; FRT82b/ FRT82b, tub-Gal80*
	B	*eyFLP.1; Act>y+>Gal4, UAS-GFP/ +; scrib1, FRT82b/ FRT82b, tub-Gal80*
	C	*eyFLP.1; Act>y+>Gal4, UAS-GFP/ +; scrib1, FRT82b, UAS-PTP10D-RNAi/ FRT82b, tub-Gal80*
	E	*eyFLP.1; Act>y+>Gal4, UAS-GFP/ +; FRT82b/ FRT82b, tub-Gal80*
	F	*eyFLP.1; Act>y+>Gal4, UAS-GFP/ +; scrib1, FRT82b/ FRT82b, tub-Gal80*

	G	*eyFLP.1; Act>y+>Gal4, UAS-GFP/ +; scrib1, FRT82b, UAS-PTP10D-RNAi/ FRT82b, tub-Gal80*
	I	*eyFLP.1; Act>y+>Gal4, UAS-GFP/ +; FRT82b/ FRT82b, tub-Gal80*
	J	*eyFLP.1; Act>y+>Gal4, UAS-GFP/ +; scrib1, FRT82b/ FRT82b, tub-Gal80*
	K	*eyFLP.1; Act>y+>Gal4, UAS-GFP/ +; scrib1, FRT82b, UAS-PTP10D-RNAi/ FRT82b, tub-Gal80*
S1	A	*eyFLP.1; Act>y+>Gal4, UAS-GFP/ +; scrib1, FRT82b/ FRT82b, tub-Gal80*
	B	*eyFLP.1; Act>y+>Gal4, UAS-GFP/ +; scrib1, FRT82b, UAS-PTP10D-RNAi/ FRT82b, tub-Gal80*
	C	*eyFLP.1; Act>y+>Gal4, UAS-GFP/ +; scrib1, FRT82b/ FRT82b, tub-Gal80*
	D	*eyFLP.1; Act>y+>Gal4, UAS-GFP/ +; scrib1, FRT82b, UAS-PTP10D-RNAi/ FRT82b, tub-Gal80*
S2	A	*FRT19a/ hsFLP, FRT19a, tub-Gal80; Act-Gal4, UAS-GFP/+; +/ +*
	B	*dlg-m52, FRT19a/ hsFLP, FRT19a, tub-Gal80; Act-Gal4, UAS-GFP/+; +/ +*
	C	*dlg-m52, FRT19a/ hsFLP, FRT19a, tub-Gal80; Act-Gal4, UAS-GFP/+; UAS-PTP10D-RNAi/ +*
	E	*UAS-GFP, hsFLP; FRT40a/ tub-Gal80, FRT40a; tub-Gal4/+*
	F	*UAS-GFP, hsFLP; lgl-27S3, FRT40a/ tub-Gal80, FRT40a; tub-Gal4/+*
	G	*UAS-GFP, hsFLP; lgl-27S3, FRT40a/ tub-Gal80, FRT40a; tub-Gal4/ UAS-PTP10D-RNAi*
	I	*FRT19a/ hsFLP, FRT19a, tub-Gal80; Act-Gal4, UAS-GFP/+; +/ +*
	J	*dlg-m52, FRT19a/ hsFLP, FRT19a, tub-Gal80; Act-Gal4, UAS-GFP/+; +/ +*
	K	*dlg-m52, FRT19a/ hsFLP, FRT19a, tub-Gal80; Act-Gal4, UAS-GFP/+; UAS-PTP10D-RNAi/ +*
	M	*hsFLP; +/ +; Act>CD2>Gal4, UAS-RFP/ +*
	N	*hsFLP; UAS-dlg-RNAi/ +; Act>CD2>Gal4, UAS-RFP/ +*
	O	*hsFLP; UAS-dlg-RNAi/ +; Act>CD2>Gal4, UAS-RFP/ UAS-PTP10D-RNAi*
	Q	*hsFLP; UAS-dlg-RNAi/ +; Act>CD2>Gal4, UAS-RFP/ +*
	R	*hsFLP; +/ +; Act>CD2>Gal4, UAS-RFP/ +*
	S	*hsFLP; UAS-scrib-RNAi/ UAS-scrib-RNAi; Act>CD2>Gal4, UAS-RFP/ +*
	T	*hsFLP; UAS-scrib-RNAi/ UAS-scrib-RNAi; Act>CD2>Gal4, UAS-RFP/ UAS-PTP10D-RNAi*
S3	A	*+/ +; tGPH/ tGPH*

Biology Open • Supplementary information

	B	*+/ +; tGPH/ tGPH*
	D	*ptc-Gal4, tub-Gal80TS/ +; +/ +*
	E	*ptc-Gal4, tub-Gal80TS/ +; UAS-dlg-RNAi/ +*
	F	*ptc-Gal4, tub-Gal80TS/ UAS-grnd-RNAi; UAS-dlg-RNAi/ +*
	H	*ptc-Gal4, tub-Gal80TS/ +; +/ +*
	I	*ptc-Gal4, tub-Gal80TS/ +; UAS-dlg-RNAi/ +*
	J	*ptc-Gal4, tub-Gal80TS/ UAS-grnd-RNAi; UAS-dlg-RNAi/ +*
	L	*ptc-Gal4, tub-Gal80TS/ +; +/ +*
	M	*ptc-Gal4, tub-Gal80TS/ +; UAS-dlg-RNAi/ +*
	N	*ptc-Gal4, tub-Gal80TS/ UAS-grnd-RNAi; UAS-dlg-RNAi/ +*
	P	*ptc-Gal4, tub-Gal80TS/ +; +/ +*
	Q	*ptc-Gal4, tub-Gal80TS/ +; UAS-dlg-RNAi/ +*
	R	*ptc-Gal4, tub-Gal80TS/ UAS-grnd-RNAi; UAS-dlg-RNAi/ +*
S4	A	*eyFLP.1; Act>y+>Gal4, UAS-GFP/ +; FRT82b/ FRT82b, tub-Gal80*
	B	*eyFLP.1; Act>y+>Gal4, UAS-GFP/ +; scrib1, FRT82b/ FRT82b, tub-Gal80*
	C	*eyFLP.1; Act>y+>Gal4, UAS-GFP/ +; FRT82b/ FRT82b, tub-Gal80, sas-eld-4*
	D	*eyFLP.1; Act>y+>Gal4, UAS-GFP/ +; scrib1, FRT82b/ FRT82b, tub-Gal80, sas-eld-4*
	G	*eyFLP.1; Act>y+>Gal4, UAS-GFP/ +; scrib1, FRT82b/ FRT82b, tub-Gal80*
	H	*eyFLP.1; Act>y+>Gal4, UAS-GFP/ +; scrib1, FRT82b/ FRT82b, tub-Gal80, sas-eld-4*

Biology Open • Supplementary information
